# Environmental Correlates of Reaching a Centenarian Age: Analysis of 144,665 Deaths in Washington State for 2011−2015

**DOI:** 10.3390/ijerph17082828

**Published:** 2020-04-20

**Authors:** Rajan Bhardwaj, Solmaz Amiri, Dedra Buchwald, Ofer Amram

**Affiliations:** 1 Elson S. Floyd College of Medicine, Washington State University, Spokane, WA 99202, USA; rajan.bhardwaj@wsu.edu; 2 Department of Nutrition and Exercise Physiology, Elson S. Floyd College of Medicine, Washington State University, Spokane, WA 99202, USA; solmaz.amiri@wsu.edu; 3 Institute for Research and Education to Advance Community Health, Elson S. Floyd College of Medicine, Washington State University, Spokane, WA 99202, USA; dedra.buchwald@wsu.edu; 4 Paul G. Allen School for Global Animal Health, Washington State University, Pullman, WA 99164, USA

**Keywords:** centenarian, mortality, aging, Washington State, social determinants

## Abstract

*Objective:* This study examined the association of several social and environmental factors on the likelihood of reaching centenarian age for older adults in Washington State. *Methods:* A survival analysis of reaching centenarian age for older adults aged 75 years and above was performed using Washington State mortality data from 2011−2015. Models were adjusted for sex, race, education, marital status, and neighborhood level social and environmental variables at the block group level. Geographic clusters of increased chance of becoming a centenarian were also mapped. *Results:* In the adjusted model, increased neighborhood walkability, lower education level, higher socioeconomic status, and a higher percent of working age population were positively associated with reaching centenarian age. Being widowed, divorced/separated, or never married were also positively correlated compared to being married. Additionally, being white or female were positively correlated with reaching centenarian status. *Discussion:* Several social and environmental factors are correlated with becoming a centenarian in Washington State. In this study, we explore findings that are consistent with previous research, as well as some that have not been previously explained. More research is needed to expand upon these findings in this rapidly growing field.

## 1. Introduction

The world was home to nearly half a million centenarians in 2015, and this number is expected to increase to around 3.7 million by 2050 [1]. In 2014, an estimated 72,197 centenarians were alive in 2014, up 43.6% since 2000 [2]. In recent years, studies on the factors influencing survival to 100 years have increased dramatically. However, several gaps in our knowledge persist regarding the demographic, environmental, and social contributors to becoming a centenarian. These gaps include factors such as walkability, access to medical care, the percentage of working age population by neighborhood, and others.

The survival probability of becoming a centenarian has been shown to be multifactorial. The rapid increase in the odds of living to 100 years of age is largely due to substantial advancements in medicine and public health that decreased the burden of disease [3,4]. Genetic factors, including genes in several pathways influencing longevity, such as inflammation and immunity, have also been explored [5]. These studies have shown that longevity is likely to be a polygenic trait, but aging has been attributed to be only 20–35% heritable [5,6]. Social and environmental factors, such as high educational attainment and socioeconomic status, also significantly contribute to longevity [4].

Healthy aging, defined by the World Health Organization “as the process of developing and maintaining the functional ability that enables wellbeing in older age,” has been shown to be an important determinant of becoming a centenarian [7]. Globally, factors such as socioeconomic status, proper sanitation, access to healthcare, public health expenditures, and others all contribute to healthy aging [8]. Studies have found that variables such as being female, being married, living in urban areas, higher educational status, lower perceived bias against oneself, social participation, and others are associated with later mortality [9,10]. For example, the survival probability of becoming a centenarian for adults aged 70 in 32 different countries was strongly correlated with four socioeconomic indicators, including gross national income, and residence in certain nations such as Japan and Canada [8].

This study aimed to examine the likelihood of becoming a centenarian for adults aged 75 and above in Washington State and to identify social and environmental correlates of healthy aging and longevity. In addition, we identified geographic clusters within Washington State where individuals are more likely to become centenarians.

## 2. Methods

Registered deaths for the years 2011–2015 were obtained from the Washington State Department of Health, Center for Health Statistics. The data file included information regarding the decedent’s sex, race, education, marital status, and residential location at the time of death (latitude and longitude) [11]. Our sample was comprised of decedents aged 75 years and older. Excluding participants under this age in our analyses eliminated the influence of some factors that commonly affect death at earlier ages, including causes of infant mortality, accidents, and congenital health conditions. Additionally, factors strongly related to mortality such as income and access to healthcare, are affected for all those above this age due to the qualification for services such as Social Security and Medicare. It is likely that individuals in this age group also are more likely to settle in a neighborhood and take advantage of the services those areas have to offer [12,13,14].

This research did not involve living human participants. As all participants were deceased, Washington State University’s Institutional Review Board waived the requirement for informed consent.

### 2.1. Outcome Variable

A time variable (years of potential life lost) and a status indicator (centenarian versus noncentenarian) worked together to define our outcome [15]. The years of potential life lost were calculated by subtracting the age at the time of death from a predetermined endpoint age. In our analyses, the endpoint age was 100, representing becoming a centenarian. Using the endpoint age of 100, an individual who died at the age of 75 had 25 years of potential life lost, and a person dying at the age of 95 had 5 years of potential life lost. The status indicator was a binary variable that distinguished between individuals who became a centenarian and those that did not.

### 2.2. Explanatory Variables

#### 2.2.1. Demographics Variable

Demographic variables were obtained from death certificates and were defined as sex (male, female), race (white, nonwhite), marital status (four categories: Married or living with domestic partner, never married, divorced or separated, or widowed), and education (four categories: No high school diploma, high school graduate or equivalent, some college, and associate’s degree or above).

#### 2.2.2. Contextual Variables

We used the area deprivation index (ADI) as a measure of socioeconomic disadvantage in neighborhoods based on 17 census variables representing the social and economic characteristics of neighborhoods in the U.S. [16,17]. This index was developed using the 2015 American Community Survey five-year estimates at the block group level. The block group is the smallest geographic unit for which the U.S. Census Bureau publishes demographic and socioeconomic data and generally contains between 600 and 3000 people, with an optimum size of 1500 people. The ADI yields scores from 1 to 10, with higher scores indicating greater socioeconomic status disadvantage [18].

The Smart Location Mapping Data provided by the Environmental Protection Agency provides an array of contextual variables at the block group level for the year 2013. Three variables from the dataset were extracted for each location of death. First, the total population able to access the block group within a 45-min transit and walking commute as a percentage of the total regional population (% population with access to transit, a continuous variable). Second, the Walkability Index based on built environment characteristics that influence the likelihood of walking being used as a mode of travel (Walkability, a continuous variable). Third, the percent of the working-age population, which provides a measure of the age diversity in block groups by measuring the percentage of the population between the ages 15−64 (% working age, a continuous variable) [19].

Access to primary care physicians (PCPs) was measured using a two-step floating catchment area as a special case of the gravity models. This approach accounts for block group population, supply of PCPs, and travel time between PCPs and block groups with a distance decay function [20]. The data regarding the supply of Washington State physicians were based on the American Medical Association Physician Masterfile, which catalogs the education, training, and professional certification on virtually all Doctors of Medicine and Doctors of Osteopathic Medicine in the United States [21]. Access scores represent a ratio of PCPs (supply) to the population of block groups served (demand), with only selected PCPs and block group population entering the numerator and denominator [22].

The Rural-Urban Commuting Area (RUCA) codes were used for delineating between metropolitan, micropolitan, or small town or rural areas. RUCA codes use work commuting information, population data, and measures of urbanization to classify U.S. Census tracts to urban and rural areas [23]. RUCA codes were developed by a collaborative between Health Resources and Service Administration’s Office of Rural Health Policy, the Department of Agriculture’s Economic Research Service, and the Washington, Wyoming, Alaska, Montana, Idaho Rural Health Research Center. RUCA primary codes of 1−3 were classified as metropolitan areas, RUCA primary codes of 4−6 were classified as micropolitan areas, and RUCA primary codes of 7−10 were classified as small towns or rural areas. A RUCA code was assigned to each individual based on their place of residence.

A PM2.5 land use regression model created in 2013 for Washington State was used to estimate air pollution exposure at each residential location (continuous variable). PM2.5 refers to particulate matter (a mixture of solid particles and liquid droplets found in the air) that are generally 2.5 micrometers and smaller [24]. Land use regression modeling is used extensively to estimate air pollution exposure using geographical information systems together with statistical analyses to predict pollution concentrations at unmeasured locations based on data measured at known locations.

The Normalized Difference Vegetation Index (NDVI) as a measure of green space exposure was calculated using the Moderate Resolution Imaging Spectroradiometer (MODIS) satellite images to assess green space exposure. Green space refers to land that is partly of completely covered with grass, trees, shrubs, or other vegetation [25]. Google Earth’s engine was used to generate average NDVI measures from satellite images at a 1000-m buffer around each residential location one year prior to the date of death (continuous).

## 3. Statistical Analysis

Univariate analyses included descriptive statistics with measures of central tendency and variability for continuous variables and frequency distributions and percentages for categorical variables. The chi-square tests were used to evaluate the associations between categorical variables. The Mann–Whitney U test was used to assess the associations between the categorical outcome and continuous explanatory variables. Kaplan-Meier and Cox regression within the family of survival analysis was used to model time-to-event data in the presence of censored cases. Kaplan-Meier with log-rank test was used to explore whether the survival times differed between white and nonwhite and female and male decedents. The unadjusted and adjusted Cox proportional hazard model was applied to examine the effect of explanatory variables on becoming a centenarian [26,27]. The effect size was presented as hazard ratios (HRs) with 95% confidence intervals (CI). The HR indicates the relative likelihood of becoming a centenarian at any given point in time, reflecting the total number of events and the timing of events. HRs greater than one signify increased likelihood of becoming a centenarian and HRs lower than one indicate that likelihood is decreased. The data were analyzed using R (company, city). The significance level was set at 0.05 (two-tailed).

### Spatial Analysis and Mapping

To identify areas of a high likelihood of reaching centenarian age, we first subtracted 100 (i.e., centenarian age) from an individual’s age at the time of death. Individuals who passed away before 100 years of age had a positive value and those who lived beyond 100 years of age had a negative value. We averaged the positive and negative values by census tract to estimate the likelihood of reaching a centenarian age by tract. Census tracts where individuals were less likely to reach 100 years of age had larger values and tracts, whereas individuals were more likely to reach 100 years of age had smaller values. Using this measure, we performed a cluster analysis using the local Getis G function on the ESRI ArcGIS 10.6 platform (ESRI, Redlands, CA, US) [28]. Getis G is a function commonly used to quantify and assess links between spatially distributed variables and to identify spatial clusters.

## 4. Results

A total of 144,665 all-cause mortality deaths at age 75 and older were reported to the Washington State Department of Health, Center for Health Statistics. Of those, 56% (*n* = 81,168) were female and 93% (*n* = 134,414) were white. At the time of death, approximately 53% (*n* = 76,575) were widowed, 33% (*n* = 47,996) were married, and the rest were divorced/separated (11%, *n* = 15771) or had never been married (3%, *n* = 3951). Most decedents (57%, *n* = 82,082) had a high school diploma, 26% (*n* = 36,915) had an associate degree or above and the rest (17%, *n* = 24,094) did not have a high school diploma or equivalent. The average age of our sample was 86 years old with only 2698 (1.8%) reaching a centenarian age (Table 1). The oldest centenarian in Washington State died at the age of 114.

The unadjusted model indicates that being male or nonwhite were risk factors for becoming a centenarian. Individuals who were widowed, never married, or divorced/separated were more likely to become centenarians compared to those who were married at the time of death. People who had a high school diploma or university degree were less likely to become centenarians compared to those with no high school diploma or equivalent. Protective factors for becoming a centenarian were greater access to public transit, higher walkability index, higher access to PCPs, and living in areas with higher percentage of working age population (HR = 15.18, 95% CI = 6.69−34.45, *p* < 0.001). Area-level deprivation and green space were negatively associated with the likelihood of becoming a centenarian. Higher PM 2.5 concentration was positively the likelihood of becoming a centenarian.

In the adjusted model (Table 2), being male or nonwhite were factors for becoming a centenarian. Individuals who were never married, divorced/separated, or widowed were more likely to become centenarians compared to those who were married at the time of death. Similar to the unadjusted results, people who had a high school diploma or university degree were less likely to become centenarians compared to those with no high school diploma. Protective factors for becoming a centenarian were higher walkability index and living in areas with a higher percentage of working age population. Area-level deprivation was negatively associated with the likelihood of becoming a centenarian. Individuals living in small towns or rural areas were less likely to become a centenarian compared to those living in metropolitan areas. Other variables were not significantly related to the likelihood of becoming centenarian in the adjusted model.

**Figure 1:**
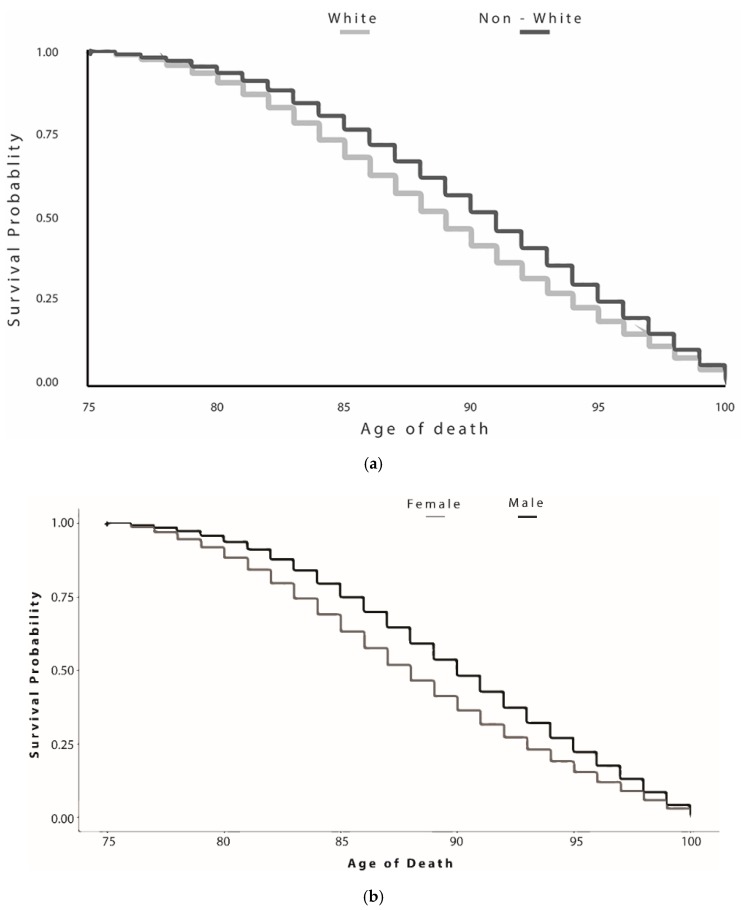
Survival curves for reaching a centenarian age among decedents in Washington State between 2011 and 2015 by race (**a**) and gender (**b**).

Figure 1 shows survival curves for reaching a centenarian age among decedents in Washington State between 2011 and 2015 by gender and by race. The hazard ratio was higher for males compared to females and for nonwhites compared to whites.

**Figure 2:**
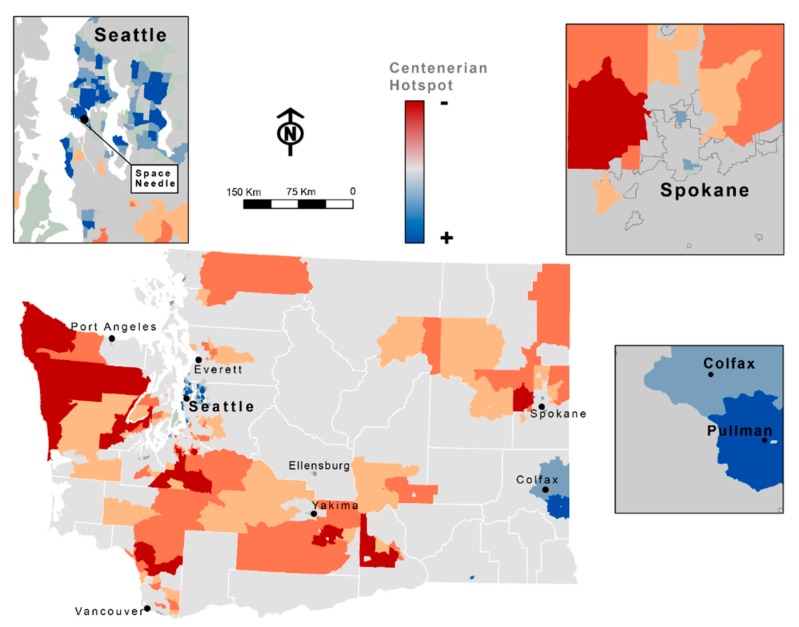
Spatial clusters of census tracts where individuals had higher (blue colors) or lower (orange and red colors) prevalence of reaching a centenarian age in Washington State between 2011 and 2015.

Figure 2 presents spatial clusters of census tracts where individuals had higher or lower prevalence of reaching a centenarian age in Washington State between 2011 and 2015. Clusters showing higher prevalence of reaching a centenarian age were primarily located in urban, higher socioeconomic census tracts in the greater Seattle area and smaller towns with higher socioeconomic census tracts in the greater Pullman region. Clusters showing lower prevalence of reaching a centenarian age were in the more rural census tract of Washington State.

## 5. Discussion

This study explored several social and environmental factors and their effects on reaching centenarian age in Washington State. We found that neighborhood walkability, education level, marital status, sex, socioeconomic status, and the percent of the population that was of working age within block groups are all associated with the likelihood of reaching a centenarian age.

Walkability had a strong positive correlation with the likelihood of reaching centenarian age by area. Walkability data have generally shown that the combination of the density of walkable intersections, dwelling density, and mixed-use land are correlated with increased walking behavior [29]. People living in walkable neighborhoods usually have easy access to public transit, healthy food, clinics/hospitals, and other services [30,31,32]. More walkable areas allow people to walk and bike for transportation and recreational purposes, which, in turn, promotes accumulating savings and physical activity [33,34,35,36,37]. In fact, walkability has been directly associated with populations with lower body mass index and other measures of health [34,38,39,40]. Walkable and bikeable streets and clean, accessible parks are linked to increasing physical activity of the surrounding population by 30% [41]. Walkable neighborhoods are especially important for older adults who may have decreased mobility and no longer drive, as they are likely to benefit from easier access to their community afforded by walkable neighborhoods [31].

Surprisingly, education was found to be negatively associated with becoming a centenarian. In recent studies, higher education levels have been strongly associated with lower mortality [42,43,44]. Higher academic level indicates employment opportunities and lifestyles associated with factors such as socioeconomic status, social connections, availability and knowledge of health resources, health behaviors (e.g., not smoking), and critical thinking skills applied to managing health problems [42,45,46]. An analysis of the U.S. National Longitudinal Mortality Study found a lower association between educational attainment and mortality among those age 65 and older compared to their counterparts ages 25−64 [43]. The authors posited that the decreasing effects of mortality with age resulted from a decreased impact of social stratification systems and education level and a greater effect of biological aging [43]. Congruent with our findings, an older study reported little to no association between educational attainment and mortality for men and women of all ages in the U.S. [47].

Furthermore, rapid advances in educational attainment in the last few generations may explain, in part, the lack of a positive association between educational attainment and becoming a centenarian in our study. In this regard, in 1950, only 34.3% of the U.S. population above the age of 25 had a high school diploma, a figure that increased to more than 80% by 2000 [44]. The high levels of educational attainment might lead to greater differences in longevity among the younger population due to the rapidly changing technological landscape of life in the U.S. and in healthcare. More recent studies have demonstrated increasing declines in mortality with education [48,49,50], suggesting that education is less of a factor in determining longevity in older populations.

Another unexpected finding was that compared to married older adults, those who never married, or were widowed, or divorced/separated were more likely to become centenarians. Being widowed showed the greatest benefit, with never having married coming second, and being divorced/separated showing the least benefit. Decades of work have consistently observed that marriage is associated with longer survival than being divorced or never having married [51,52,53,54,55]. These papers suggest the theory of “marriage protection,” which refers to the environmental, social, and psychological factors that make being married healthier. For example, being married is associated with greater social connectedness, which has also been independently associated with later mortality [52,54]. It has also been proposed that not being married reflects a process referred to as “marital selection,” where those who do not marry are inherently less healthy [53,56].

However, this study specifically focused on those aged 75 and above, so the selection aspect and some of the protective factors may not be as relevant. Many studies have not explored the effects of marital status on health at older ages specifically. One study focusing on the elderly found that being a widow/widower or a single man was associated with poorer health, but single women were likely to live longer [54]. A significant negative effect of marital dissolution has generally been found in men but not women [57]. In this study, the finding of a much greater likelihood of becoming a centenarian for those who are widowed may be partially explained by the fact that those who lost their spouses earlier in life may no longer experience the stresses associated with the traumatic event. The death of a spouse at a young age involves a more substantial restructuring of life, including single parenthood [57]. Additionally, strained marriages may in and of themselves cause increased stress and diminished health outcomes. This line of reasoning may also contribute to the findings around being divorced/separated leading to a greater likelihood of becoming a centenarian, which is not generally consistent with prior research [56]. Those who were widowed, never married, or divorced/separated may not have had to face the consequences of unhealthy marriages. More research is likely necessary to explain these findings.

This study found that higher ADI was associated with a lower likelihood of becoming a centenarian. Socioeconomic status was also strongly linked to the likelihood of reaching centenarian age. Several measures of socioeconomic status have been tested and used to confirm this finding [8,58,59,60]. Income itself has also been related directly to health [61,62,63]. The rationale behind these findings, like for education, relate to healthier lifestyle choices (not smoking, physical activity, etc.), being able to afford health interventions, having social connections, and others [64,65]. Higher ADI specifically has also been previously linked to increasing mortality in the general U.S. population [16].

It is well known that women in the U.S. and worldwide are more likely than men to live longer and reach centenarian status [8,10]. This study found that the probability of becoming a centenarian was significantly smaller for males than females. The explanations for this finding are not completely known but range from biological differences between women and men to social and environmental factors such as healthy behaviors [66,67,68]. Nonwhite individuals were also less likely to reach a centenarian age compared to white individuals. Research in the U.S. has consistently found higher mortality rates for African Americans compared to white Americans at all ages except for a crossover at very old ages [69,70,71]. Conversely, Hispanics and Asians/Pacific Islanders have been shown to have lower mortality rate compared to white individuals at all ages [69,70,71]. The data in this study were not stratified by specific racial groups, so the differences in mortality among these groups cannot be determined. The explanation for this finding may be related to the effects of discrimination, lower socioeconomic status, poorer health outcomes, etc. [10,71].

Lastly, the percent of the working age population was correlated with a higher likelihood of reaching centenarian age. This indicator measures the percent of individuals 15−64 that are employed. It is well known that more urban communities are more likely to have younger populations and more labor force participants, which suggests higher educational attainment and earnings [72]. Higher percentages of working age populations are seen in more urban areas due to the greater availability of work, easier access to services and programs, and a preference for the lifestyle. Studies have consistently found higher mortality rates in rural vs. urban areas [73,74]. Interestingly, however, our variable indicating rural-urban differences was not significant. The movement of younger people from rural to more urban areas results in less support and services for the elderly that are left in those communities. Communities with higher working age populations have higher socioeconomic status, more government support, and better access to transportation and healthcare services [72,74]. These factors have been shown separately to influence longevity and the chance of becoming a centenarian.

The limitations of this study include that it is an observational study. The factors affecting mortality are complex, and it is difficult to know which variables may have the greatest effect on subjects without conducting longitudinal studies and without actual interventions. Also, we did not have information on where individuals lived for their entire lives. The home address used in this study were based on their address at the time of death. As a result, it is impossible to know how the previous communities’ subjects inhabited influenced their longevity. Recent years of research into centenarians have raised many interesting findings. However, there are still many areas of the intersection between mortality, centenarians, and social determinants of health to explore.

## 6. Conclusions

This study demonstrated that several social and environmental factors were associated with becoming a centenarian in Washington State based on mortality data from 2011−2015 for individuals over 75. These factors included neighborhood walkability, education level, marital status, sex, socioeconomic status, and the percent of the population that was of working age. More research into this important subject is required.

## Figures and Tables

**Table 1:** Characteristics of decedents in Washington State between 2011 and 2015 (*n* = 144,665), overall and stratified by reaching a centenarian age.

Characteristics	All Decedents (144,665 (98.1%))	75−99 Years Old (141,967 (98.1%))	≥100 Years Old (2698 (1.8%))	*p*-Value
*Demographic Variables*				
Age (Median (IQR)	86 (81−91)	86 (81−91)	101 (100−102)	<0.001
Gender (no. (%))				
Female	81,168 (56.1)	79,026 (55.7)	2142 (79.4)	<0.001
Male	63,497 (43.9)	62,941 (44.3)	556 (20.6)	
Race (no. (%))				
Nonwhite	9873 (6.8)	9721 (6.8)	152 (5.6)	0.01
White	134,414 (92.9)	131,872 (92.9)	2542 (94.2)	
NA	378 (0.3)	374 (0.3)	4 (0.1)	
Marital Status (no. (%))				
Married	47,996 (33.2)	47,877 (33.7)	119 (4.4)	<0.001
Never Married	3951 (2.7)	3878 (2.7)	73 (2.7)	
Divorced or Separated	15,771 (10.9)	15,660 (11)	111 (4.1)	
Widowed	76,575 (52.9)	74,185 (52.3)	2390 (88.6)	
NA	372 (0.3)	367 (0.3)	5 (0.2)	
Education Level (no. (%))				
No High School Diploma	24,094 (16.7)	23,487 (16.5)	607 (22.5)	<0.001
High School Diploma	82,082 (56.7)	80,660 (56.8)	1422 (52.7)	
Associate Degree or Above	36,915 (25.5)	36,273 (25.6)	642 (23.8)	
NA	1574 (1.1)	1547 (1.1)	27 (1)	
*Contextual Variables*				
Urban vs. Rural (no. (%))				
Metropolitan Area	122,314 (84.5)	120,031 (84.5)	2283 (84.6)	0.96
Micropolitan Area	12,235 (8.5)	12,005 (8.5)	230 (8.5)	
Small Town or Rural	10116 (7)	9931 (7)	185 (6.9)	
Access to Public Transportation (Median (IQR)	0 (0−0.06)	0 (0−0.06)	0.02 (0−0.08)	<0.001
Walkability (Median (IQR)	9.83 (6.7−14)	9.67 (6.5−14)	10.83 (7.5−14.7)	<0.001
Area deprivation index (Median (IQR)	6 (3−8)	6 (3−8)	5 (3−8)	<0.001
Access to primary care Physicians (Median (IQR)	9.42 (6.9−12)	9.41 (6.9−12)	9.8 (7.1−12.4)	<0.001
Percent of Working Age (Median (IQR)	0.78 (0.8−0.8)	0 (0−0.06)	0.79 (0.8−0.8)	<0.001
Greenspace (NDVI) (Median (IQR)	0.52 (0.4−0.6)	0.52 (0.4−0.6)	0.5 (0.4−0.6)	<0.001
Air Pollution (PM 2.5) (Median (IQR)	6.6 (5.9−7.4)	6.5 (5.9−7.4)	6.7 (6−7.5)	<0.001

NOTE: IQR: Interquartile range; NDVI: Normalized difference vegetation index; PM: Particulate matter.

**Table 2:** Adjusted analyses of characteristics associated with reaching a centenarian age among decedents in Washington State between 2011 and 2015.

Characteristics	Unadjusted HR (95%CI)	*p*-value	Adjusted HR (95%CI)	*p*-Value
*Demographic Variables*				
Gender				
Female	Reference		Reference	
Male	0.33 (0.30−0.36)	<0.001	0.56 (0.50−0.63)	<0.001
Race				
White	Reference		Reference	
Nonwhite	0.81 (0.67−0.98)	0.03	0.71 (0.58−0.87)	<0.001
Marital Status				
Married	Reference		Reference	
Never Married	7.51 (5.61−10.06)	<0.001	6.44 (4.68−8.85)	<0.001
Divorced or Separated	2.85 (2.18−3.71)	<0.001	2.53 (1.89−3.39)	<0.001
Widowed	12.77 (10.64−15.34)	<0.001	9.72 (7.90−11.96)	<0.001
Education Level				
No High School Diploma	Reference		Reference	
High School Diploma	0.68 (0.62−0.75)	<0.001	0.61 (0.55−0.68)	<0.001
Associate Degree or Above	0.69 (0.61−0.77)	<0.001	0.73 (0.64−0.84)	<0.001
*Contextual Variables*				
Urban Versus Rural				
Metropolitan Area	Reference		Reference	
Micropolitan Area	1.01 (0.87−1.17)	0.9	0.93 (0.74−1.17)	0.5
Small Town or Rural	0.98 (0.82−1.16)	0.8	0.43 (0.21−0.88)	0.02
Access to Public Transportation	2.14 (1.09−4.23)	0.03	0.73 (0.33−1.58)	0.4
Walkability	1.04 (1.03−1.06)	<0.001	1.03 (1.01−1.05)	<0.001
Area Deprivation Index (ADI)	0.97 (0.96−0.99)	0.006	0.96 (0.94−0.98)	<0.001
Access to Primary Care Physicians	1.04 (1.02−1.05)	<0.001	0.99 (0.96−1.01)	0.3
Percent Working Age	15.18 (6.69−34.45)	<0.001	13.02 (5.28−32.09)	<0.001
Greenspace (NDVI)	0.47 (0.33−0.66)	<0.001	0.85 (0.48−1.53)	0.6
Air Pollution (PM2.5)	1.07 (1.03−1.12)	0.002	0.99 (0.93−1.05)	0.6

HR indicates the relative likelihood of becoming a centenarian at any given point in time, reflecting the total number of events and the timing of events.
